# Long-Term Upregulation of Inflammation and Suppression of Cell Proliferation in the Brain of Adult Rats Exposed to Traumatic Brain Injury Using the Controlled Cortical Impact Model

**DOI:** 10.1371/journal.pone.0053376

**Published:** 2013-01-03

**Authors:** Sandra A. Acosta, Naoki Tajiri, Kazutaka Shinozuka, Hiroto Ishikawa, Bethany Grimmig, David Diamond, Paul R. Sanberg, Paula C. Bickford, Yuji Kaneko, Cesar V. Borlongan

**Affiliations:** 1 Center of Excellence for Aging and Brain Repair, Department of Neurosurgery and Brain Repair, University of South Florida College of Medicine, Tampa, Florida, United States of America; 2 James A. Haley Veterans Affairs Hospital, Tampa, Florida, United States of America; 3 Department of Psychology, University of South Florida, Tampa, Florida, United States of America; 4 Office of Research and Innovation, University of South Florida, Tampa, Florida, United States of America; University of Victoria, Canada

## Abstract

The long-term consequences of traumatic brain injury (TBI), specifically the detrimental effects of inflammation on the neurogenic niches, are not very well understood. In the present in vivo study, we examined the prolonged pathological outcomes of experimental TBI in different parts of the rat brain with special emphasis on inflammation and neurogenesis. Sixty days after moderate controlled cortical impact injury, adult Sprague-Dawley male rats were euthanized and brain tissues harvested. Antibodies against the activated microglial marker, OX6, the cell cycle-regulating protein marker, Ki67, and the immature neuronal marker, doublecortin, DCX, were used to estimate microglial activation, cell proliferation, and neuronal differentiation, respectively, in the subventricular zone (SVZ), subgranular zone (SGZ), striatum, thalamus, and cerebral peduncle. Stereology-based analyses revealed significant exacerbation of OX6-positive activated microglial cells in the striatum, thalamus, and cerebral peduncle. In parallel, significant decrements in Ki67-positive proliferating cells in SVZ and SGZ, but only trends of reduced DCX-positive immature neuronal cells in SVZ and SGZ were detected relative to sham control group. These results indicate a progressive deterioration of the TBI brain over time characterized by elevated inflammation and suppressed neurogenesis. Therapeutic intervention at the chronic stage of TBI may confer abrogation of these deleterious cell death processes.

## Introduction

In the United States alone, an estimated 1.7 million people suffer from traumatic brain injury (TBI), and nearly 52,000 deaths a year, accounting for 30% of all injury-related deaths [1]. Annually, the cost of TBI related expenses is estimated to be around 52 billion dollars [2], [3]. Patients who survive head injuries often present with disabilities persisting up to decades after the injury [4]. Although the severity of disabilities varies, which may be directly associated with the severity of the injury itself [5], the most common disabilities include sensory-motor problems, learning and memory deficits, anxiety, and depression [5], [6]. Notably, TBI may predispose long-term survivors to age-related neurodegenerative diseases such as Alzheimer's disease, Parkinson’s disease, and post-traumatic dementia [5], [6], [7], [8], [9], [10].

Long-term neurological deficits from TBI are associated with neuroinflammation, and may aggravate over time to more severe secondary injuries, making prevention and treatment a very complex task [1], [11], [12], [13], [14]. Currently, a very well characterized TBI model for chronic brain atrophy, which addresses proximal and distal subcortical regions vulnerable to injury, is not available. An in-depth histological examination of the brain at the chronic stage of TBI should provide insights into identifying therapeutic targets amenable to treatment interventions even when initiated at this late phase of disease progression. Unfortunately to date, many studies concentrate on specific subcortical regions, while others focus only on white matter, making it difficult to translate the findings on pathological mechanisms and therapies generated in TBI animal models to clinical applications [15], [16], [17], [18]. A better understanding of the neuropathology propagation associated with TBI, through investigations of neuro-inflammatory mechanisms will allow us to efficiently manage and treat the evolution of TBI-secondary neuropathologies and cognitive disabilities after the acute phase [11], [19]. In the present in vivo study, the neuro-inflammatory responses in subcortical regions, such as the dorsal striatum, thalamus, and white matter as corpus callosum, hippocampal fimbria-fornix, and cerebral peduncle were characterized in chronic TBI. Additionally, neuronal cell loss, cell proliferation and neuronal differentiation were examined in neurogenic niches to assess the detrimental influence of progressive secondary injury in these vital regenerative areas of the brain. Our overarching theme advances the concept that a massive neuroinflammation after TBI represents a second wave of cell death that impairs the proliferative capacity of cells, and impedes the regenerative capacity of neurogenesis in chronic TBI. Accordingly, we embarked on this study to test the hypothesis that chronic TBI-induced neuroinflammation interfered endogenous repair mechanisms.

## Materials and Methods

### Subjects

Experimental procedures were approved by the University of South Florida Institutional Animal Care and Use Committee (IACUC). All animals were housed under normal conditions (20°C, 50% relative humidity, and a 12-h light/dark cycle) All studies were performed by personnel blinded to the treatment condition.

### Surgical Procedures

Ten-week old Sprague–Dawley rats (n = 24) were subjected to either TBI using a controlled cortical impactor (CCI) (n = 12) or sham control (no TBI) (n = 12) (Pittsburgh Precision Instruments, Inc, Pittsburgh, PA). Deep anesthesia was achieved using 1–2% isoflurane, and it was maintained using a gas mask. All animals were fixed in a stereotaxic frame (David Kopf Instruments, Tujunga, CA, USA). After exposing the skull, coordinates of −0.2 mm anterior and +0.2 mm lateral to the midline were used and impacted the brain at the fronto-parietal cortex with a velocity of 6.0 m/s reaching a depth of 1.0 mm below the dura matter layer and remained in the brain for 150 milliseconds (ms). The impactor rod was angled 15° degrees vertically to maintain a perpendicular position in reference to the tangential plane of the brain curvature at the impact surface. A linear variable displacement transducer (Macrosensors, Pennsauken, NJ), which was connected to the impactor, measured the velocity and duration to verify consistency. Sham control injury surgeries consisted of animals exposed to anesthesia, scalp incision, craniectomy, and suturing. An electric drill was used to perform the craniectomy of about 2.5 mm radius with coordinates calculated from the bregma at −0.2 anterior and +0.2 mm lateral right. An automated thermal blanket pad and a rectal thermometer allowed maintenance of body temperature within normal limits. All animals were closely monitored post-operatively with weight and health surveillance recording as per IACUC guidelines. Rats were kept hydrated at all times, and the analgesic ketoprofen was administered after TBI surgery and as needed thereafter. Pre and post TBI, rats were fed regular rodent diet (Harlan 2018, Harlan).

### Hematoxylin and Eosin Analysis

Hematoxylin and eosin (H&E) staining was performed to confirm the core impact injury of our TBI model. As shown in our previous studies [3], [5], we also demonstrated here that the primary damage produced by the CCI TBI model was to the fronto-parietal cortex. In addition, H&E staining was analyzed in the hippocampus to reveal secondary cell loss. Starting at coordinates AP-2.0 mm and ending at AP-3.8 mm from bregma, coronal brain sections (40 µm) covering the dorsal hippocampus were selected. A series of 6 sections per rat was processed for staining. Cells presenting with nuclear and cytoplasmic staining (H&E) were manually counted in the CA3 neurons. CA3 cell counting spanned the whole CA3 area, starting from the end of hilar neurons to the beginning of curvature of the CA2 region in both the ipsilateral and contralateral side. Sections were examined with Nikon Eclipse 600 microscope at 20X All data are represented as mean values ±SEM, with statistical significance set at p<0.05.

### Immunohistochemistry

Under deep anesthesia, rats were sacrificed 8 weeks after TBI surgery, and perfused through the ascending aorta with 200 ml of ice cold phosphate buffer saline (PBS), followed by 200 ml of 4% paraformaldehyde (PFA) in PBS. Brains were removed and post-fixed in the same fixative for 24 hours followed by 30% sucrose in phosphate buffer (PB) for 1 week. Coronal sectioning was carried out at a thickness of 40 µm by cryostat. H&E staining was done on every sixth coronal section spanning the dorsal hippocampus. Staining for the cell cycle–regulating protein Ki67, DCX, and OX6 was done on every sixth coronal section throughout the entire striatum and dorsal hippocampus. Sixteen free-floating coronal sections (40 µm) were incubated in 0.3% hydrogen peroxide (H_2_O_2_) solution followed by 1-h of incubation in blocking solution (0.1 M phosphate-buffered saline (PBS) supplemented with 3% normal goat serum and 0.2% Triton X-100). Sections were then incubated overnight with Ki67 (1∶400 Nocastra), DCX (1∶150 Santa Cruz), and OX6 (major histocompatibility complex or MHC class II; 1∶750 BD) antibody markers in PBS supplemented with 3% normal goat serum and 0.1% Triton X-100. Sections were then washed and biotinylated secondary antibody (1∶200; Vector Laboratories, Burlingame, CA) in PBS supplemented with 3% normal goat serum, and 0.1% Triton X-100 was applied for 1 h. Next, the sections were incubated for 60 minutes in avidin–biotin substrate (ABC kit, Vector Laboratories, Burlingame, CA). All sections were then incubated for 1 minute in 3,30-diaminobenzidine (DAB) solution (Vector Laboratories). Sections were then mounted onto glass slides, dehydrated in ethanol and xylene, and cover-slipped using mounting medium.

### Stereological Analysis

Unbiased stereology was performed on brain sections immunostained with OX6, Ki67 and DCX. Sets of 1/6 section, about 240 µm apart, were taken from the brain spanning AP –0.2 mm to AP –3.8 mm in all 24 rats. Activated microglia cells, cell proliferation, and differentiation into immature neurons were visualized by staining with OX6, Ki67, and DCX, respectively. Positive stains were analyzed with a Nikon Eclipse 600 microscope and quantified using Stereo Investigator software, version 10 (MicroBrightField, Colchester, VT). The estimated volume of OX6-positive cells was examined using the Cavalieri estimator probe of the unbiased stereological cell technique [20] revealing the volume of OX6 in the cortex, striatum, thalamus, fornix, cerebral peduncle, and corpus callosum. Ki67 [21] and DCX positive cells were counted within the subgranular zone (SGZ) and the subventricular zone (SVZ), in both hemispheres (ipsilateral and contralateral), using the optical fractionator probe of unbiased stereological cell counting technique. The sampling was optimized to count at least 300 cells per animal with error coefficients less than 0.07. Each counting frame (100×100 µm for OX6, Ki67, and DCX) was placed at an intersection of the lines forming a virtual grid (125×125 µm), which was randomly generated and placed by the software within the outlined structure. Section thickness was measured in all counting sites.

### Statistical Analysis

For data analyses, contralateral and ipsilateral corresponding brain areas were used as raw data providing 2 sets of data per treatment condition (TBI vs. sham control), therefore one-way analysis of variance (ANOVA) was used for group comparisons, followed by subsequent pairwise comparisons; post hoc tests Bonferonni test. All data are represented as mean values with ±SEM. Statistical significance was set at *p*<0.05 for all analyses.

## Results

In the preliminary analyses of the data, comparisons between sham control ipsilateral and sham control contralateral side, across all brain regions studied, did not significantly differ (*p*>0.05). Thus, the data from both sides of the sham group were combined. Pair-wise comparisons are summarized in the Tables S1, S2, S3, S4, S5, S6, S7, S8, S9, including neuroinflammation in different regions of the brain (cortex, Table S1; striatum, Table S2; thalamus, Table S3; corpus callosum, Table S4; cerebral peduncle, Table S5; fornix, Table S6), CA3 neuronal cell loss (Table S7), cell proliferation in SVZ (Table S8), and cell proliferation in SGZ (Table S9).

### Upregulation of MHCll+ activated Microglia Cells in Chronic TBI

**Figure 1:**
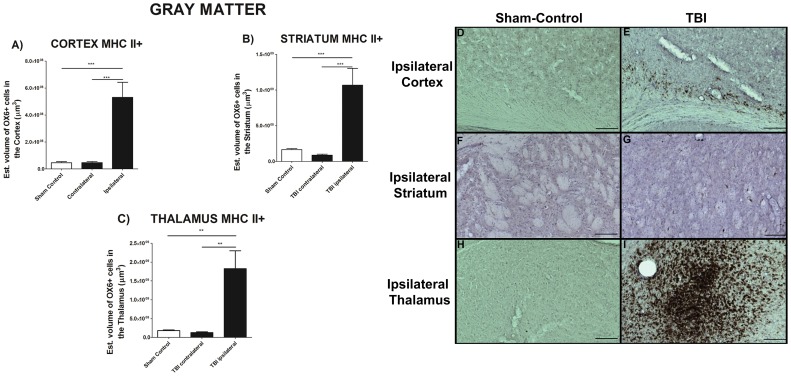
Upregulation of MHCll+ activated microglia cells in gray matter in chronic TBI. Results indicate that there is a clear exacerbation of activated microglia cells in ipsilateral side of subcortical gray matter regions in chronic TBI relative to contralateral side and sham control. After 8 weeks from initial TBI injury, asterisks denote significant upregulation on the volume of MHC II expressing cells in A) cortex, B) striatum, C) thalamus. While contralateral side present an estimated volume of activated microglia cells similar to sham control animals. ANOVA revealed significant treatment effects as follows: cortex, F_2,45_ = 18.49; ***p<0.005; striatum, F_2,45_ = 15.71, ***p<0.005, and; thalamus, F_2,45_ = 12.23, ***p<0.005. Photomicrographs correspond to representative gray matter in coronal sections stained with OX6 (MHC ll) from ipsilateral sham control and TBI rats, cortex (Figure 1D, E), striatum (Figure 1F, G), thalamus (Figure 1H, I). Scale bars for D, E, F, G, H, I = 1 µm.

**Figure 2:**
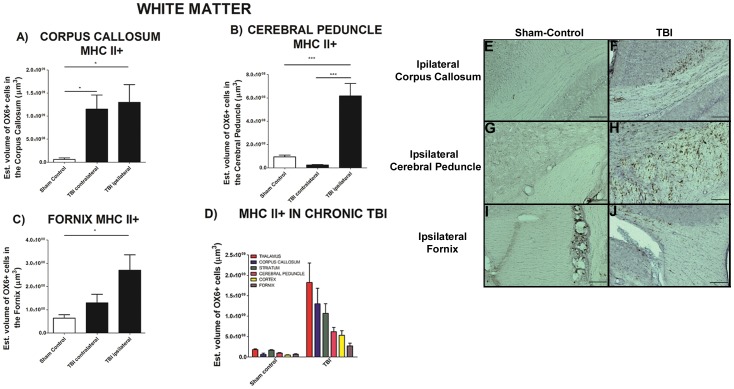
Upregulation of MHCll+ activated microglia cells in white matter in chronic TBI. Results indicate that there is an upregulation of activated microglia cells after 8 weeks post TBI in proximal white matter areas. There is an upregulation of MHCll+ cells in the ipsilateral and contralateral side of corpus callosum relative to sham control (Figure 2A). In contrast, upregulation of MHCll+ activated microglia cells in the cerebral peduncle (Figure 2B) and fornix (Figure 2C) is only present in the ipsilateral side as compared with the contralateral and sham control. There were no significant differences between contralateral side and sham control animals in (Figure 2B) and (Figure 2C). ANOVA revealed significant treatment effects as follows: corpus callosum, F_2,45_ = 5.656; *p<0.05; cerebral peduncle, F_2,45_ = 27.39, ***p<0.0005, and; fornix, F_2,45_ = 5.541, *p<0.05. Representative photomicrographs, ipsilateral corpus callosum, sham-control Figure 2E and TBI Figure 2F, ipsilateral cerebral peduncle, sham-control Figure 2G and TBI Figure 2H, and ipsilateral Fornix, sham-control Figure 2I and TBI Figure 2J. Scale bars for Figure 2E, F, G, H, I, J = 1 µm. A summary of MHCll+ estimated volume is presented capturing different subcortical regions; including those proximal and distal from TBI insult (Figure 2D). Chronic TBI greatly upregulates the neuroinflammation in the thalamus expressing the highest upregulation of MHCll+ activated microglia cells, despite its distal subcortical location. Strong expression of MHCll+ activated microglia cells is also detected in the corpus callosum and striatum (Figure 2D).

To test the hypothesis that the chronic stage of TBI was accompanied by upregulation of activated microglia cells (MHC ll+), gray and white matter areas were examined such as cortex, striatum, thalamus, olfactory bulb, dentate gyrus, corpus callosum, cerebral peduncle and fornix (Figure 1 and Figure 2). Of note, the dentate gyrus and olfactory bulbs displayed no detectable OX6-immunoreactive cells (see also Figure S1 and Figure S2). We calculated the volume of activated microglia cells (MHC ll +) in the ipsilateral and contralateral areas using an anti-OX6 antibody. Chronic TBI produced a robust upregulation in the volume of MHC II-labeled activated microglia cells in gray matter areas ipsilateral to TBI, whereas the volume in the contralateral side was not significantly different to that in sham control (Figure 1A, B, C). There was a 12-, 7- and a 10-fold increase in the volume of MHC Class ll in cortex (Figure 1A, D, E), striatum (Figure 1B, F,G), and thalamus (Figure 1C, H, I), respectively; cortex, F_2, 45_ = 18.49, *p*<0.005; striatum, F_2, 45_ = 15.71, *p*<0.005; thalamus, F_2, 45_ = 12.23, *p*<0.005. Similar analysis show that chronic TBI prompted an increase of activated MHC II-positive microglia cell volume in white matter areas ipsilateral and contralateral to TBI injury (Figure 2 and Figure S2). Chronic TBI resulted in an upregulation of activated microglia cells in corpus callosum, cerebral peduncle, and fornix around the injury side (Figure 2A, B, and C). There were no significant differences between ipsilateral and contralateral side of TBI animals, largely due to activation of microglia cells in corpus callosum in both hemispheres (*p’s*>0.05; Figure 2A). Additionally, significant increments in activated MHC II-positive microglia cells were detected in the ipsilateral cerebral peduncle (Figure 2A) and the ipsilateral hippocampal fornix (Figure 2C). ANOVA revealed significant treatment effects on MHC II-positive cells as follows: corpus callosum, F_2, 45_ = 5.656, *p*<0.05; cerebral peduncle, F_2, 45_ = 27.39, *p*<0.0005; fornix, F_2, 45_ = 5.541, *p*<0.05. A summary of all areas, comparing sham control and chronic TBI, is presented in Figure 2D. All data are represented as mean values ±SEM.

### Chronic TBI Impairs Hippocampal Cell Survival and Proliferation

**Figure 3:**
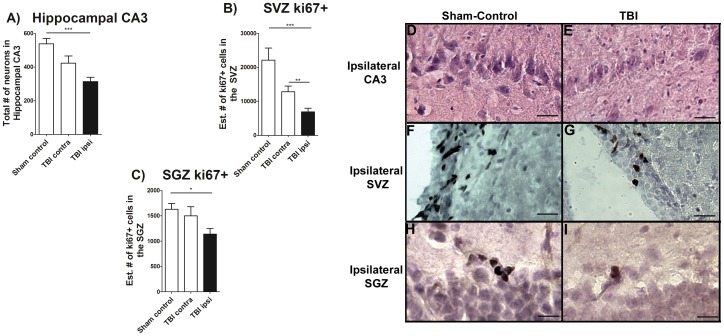
Hippocampal CA3 cell loss and downregulation of cell proliferation. H&E staining revealed a significant cell loss in the hippocampal CA3 region after chronic TBI (Figure 3A). Ki67, a cell proliferation marker, revealed a significant chronic TBI-related decrease in the SVZ of cell proliferation only in the ipsilateral side relative to contralateral side and sham control animals (Figure 3B). Contralateral measurements revealed that cell proliferation also decrease, but it does not show significant differences when compared with sham control animals (Figure 3B). Also, Ki67 revealed a significant decrease in cell proliferation in the SGZ of the hippocampus in the ipsilateral side compared to sham control (Figure 3C). In summary, ANOVA revealed significant treatment effects as follows: Hippocampal CA3 neurons, F_2,9_ = 10.78, ***p<0.005; SVZ, F_2,45_ = 10.45, ***p<0.005, and; SGZ, F_2,45_ = 3.755, ***p<0.005. Representative photomicrographs from coronal sections ipsilateral CA3 region stained with hematoxylin/eosin in sham control and TBI rats (Figure 3D, E). Ipsilateral SVZ from sham-control and TBI rats (Figure 3F, G) and ipsilateral SGZ from sham-control and TBI rats (Figure 3H, I) are shown. Scale bars for Figures 3D, E, F, G, H, I = 50 µm.

Next, in order to test the hypothesis that neuronal cell loss and impaired cell proliferation accompanied long term chronic TBI, the total number of surviving neurons in the hippocampal CA3 region, and the estimated number of positive dividing cells within SVZ and SGZ were examined. We found that long term chronic TBI significantly affected CA3 cell survival; F_2,9_ = 10.78, *p*<0.005, characterized by decreased neurons in the CA3 area of the ipsilateral hippocampus relative to sham control; p<0.05 (Figure 3A). There was no significant loss of neurons in the CA3 contralateral to chronic TBI animals compared to sham control (*p*>0.05; Figure 3A). Additionally, cell proliferation was examined by quantifying the cell proliferation marker Ki67 (Figure 3 and Figure S3). Chronic TBI significantly reduced cell proliferation in SVZ (F_2, 45_ = 10.45, *p*<0.0005) in both the ipsilateral and contralateral side compared with sham control (*p’s*<0.05; Figure 3B). Following this observation of chronic TBI-induced downregulation in the SVZ, we next inspected the cell proliferation in the SGZ, another neurogenic niche (Figure 3 and Figure S3). Again, chronic TBI was found to disturb cell proliferation in the SGZ (F_2, 45_ = 3.755, *p*<0.005). Quantification of cell proliferation within the SGZ demonstrated that there was a significant decrease in cell proliferation only in the ipsilateral side of chronic TBI compared with sham control (*p*<0.05). The contralateral SGZ did not show significant decrements in cell proliferation relative to sham control (*p*>0.05). The dentate gyrus (see Figure S3) and olfactory bulb (not shown) did not display overt cell loss.

### Chronic TBI does not Affect Neuronal Differentiation in Neurogenic Niches

**Figure 4:**
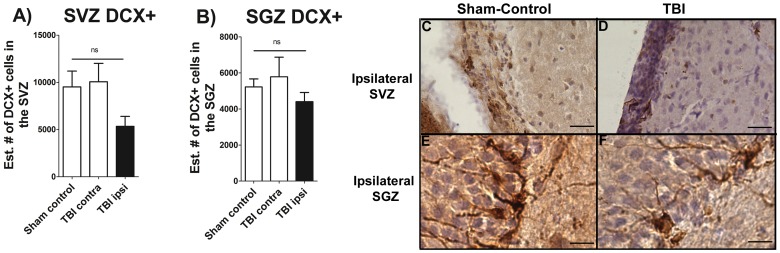
Neural differentiation is not affected by chronic TBI. DCX staining, neural differentiation marker revealed that there is not significant impairment in neural differentiation in either SVZ of the lateral ventricle, or the SGZ of the hippocampus relative to contralateral side and sham control animals. The “ns” denotes non-significant differences (p>0.05). Representative coronal sections from ipsilateral SVZ stained with DCX in sham control and TBI rats (Figure 4C, D) and SGZ from sham-control and TBI rats (Figure 4E, F) are shown. Scale bars for Figure 4C, D, E, F = 50 µm.

Since chronic TBI induced extensive downregulation of cell proliferation in the two main neurogenic niches (SVZ and SGZ), neuronal differentiation was examined. Although there appeared a general downregulation of DCX-positive cells, the fraction of new cells generated in the SVZ and SGZ initiating down the neuronal path seems to be similar in the control compared to the TBI conditions (Figure 4A and B). Chronic TBI did not significantly impair cellular differentiation into neuronal lineage in the ipsilateral SVZ and SGZ when compared with the corresponding contralateral side or with sham control animals (*p*>0.05).

## Discussion

The present study demonstrated long-term neuroinflammation accompanied chronic TBI, which was closely associated with neuronal cell loss and impaired cell proliferation in discrete brain areas adjacent to and even in remote structures from the core injured region. At eight weeks post-TBI, a significant upregulation of activated microglia cells was detected not only in the directly TBI impacted cortical site, but also in proximal adjacent ipsilateral areas as well as in distal areas from injury. In tandem, a significant decrease of hippocampal neurons in the CA3 region ipsilateral to injury was detected relative to sham control. There was no cell loss found in the contralateral side after chronic TBI in the CA3 region. Examination of the neurogenic niches revealed significant declines in cell proliferation in both SVZ and SGZ ipsilateral to TBI. Of note, only the contralateral side of SVZ, but not the SGZ, seemed to be affected by chronic TBI showing a 40% decreased in cell proliferation compared with sham control. The present location of chronic inflammation seems to correlate with the observed cell loss and impaired cell proliferation. The SVZ and the dorsal hippocampus are located proximal to the area of CCI in the cortex. In addition, the proximity of the fornix and corpus callosum and thalamus to the hippocampus might have affected the CA3 cell survival and SGZ cell proliferation due to the chronic activated microglia cells present in these regions.

Neurodegeneration after the initial insult in TBI involves acute and chronic stages. Cell death processes in acute, but not chronic stages of TBI, as revealed by the CCI model, have been well characterized [5], [22], [23], [24]. Acute primary injury manifestations start to appear during the early stages of TBI, characterized by elevated intracerebral pressure, ruptured blood brain barrier, brain edema, and reduced cerebral blood flow at the area of injury [28], [29], [30]. In addition, at the molecular level, a massive innate immune response appears within minutes to ease elimination of cellular debris [24]. This wave of progressive injury contributes to long-term progressive damage post-TBI in animals and is seen also in patients even decades after the injury [6], [8], [9], [27]. After acute head trauma, increased cell proliferation and neural differentiation were detected within the neurogenic niches (SVZ and SGZ), likely corresponding to an endogenous regenerative mechanism to provide neuroprotection at the site of injury [3], [31], [32], [33], [34], [35], [36]. The recognition that the chronic stage of neuroinflammation alters endogenous reparative mechanism, i.e., proliferative properties of neurogenic niches especially the SVZ [38], requires the development of new strategies to mobilize these proliferative cells to specific injured brain areas for regenerative purposes [38]. Of note, only 10% of new cells in the SGZ survive for up to 4 weeks post injury in a close head injury mouse model of focal TBI, and 60% of new cells in the pericontusional cortex become astrocytes in response to brain injury [32], [33]. Additionally, a decreased survival of immature neurons can be seen as early as 7 days, in a mouse model of moderate TBI, and then at 4 weeks post-TBI both cell proliferation and neural differentiation greatly declined relative to sham control mice [37]. The observed preferential effect of TBI on cell proliferation, but not neuronal differentiation, may be due to a fraction of new cells undergoing neurogenesis not affected by TBI, even though overall proliferation rates are substantially reduced. In particular, this fraction of new cells generated in the SVZ and SGZ committed towards the neuronal lineage remains active in both control and TBI conditions. The type of insult (TBI, radiation, neurotoxin) may affect the brain microenvironment with varying levels of signaling cues for cell proliferation and differentiation, in turn resulting in an imbalance of new dividing cells and cells differentiating to a neuronal phenotype. This hypothesis clearly warrants further investigations.

Progressive injury to hippocampal, cortical, and thalamic areas contributes to long-term cognitive damage post-TBI as noted in military men and in civilian patients even decades after the injury [5], [6], [8], [9], [22], [24], [25], [26], [27], [30]. It is well recognized that hippocampal cell loss is a consequence of TBI [39], [40]. TBI patients have been shown to exhibit deficits in verbal declarative memory, which is modulated in part by the hippocampal formation, and executive functioning [41], [43]. Neuropsychological tests performed in US Army soldiers after deployment revealed that TBI is closely associated with functional impairments, while TBI co-morbid with PTSD and depression presents with chronic long lasting cognitive deficits [26]. In addition, cognitive functions modulated by the thalamic-cortical areas of the brain are also affected by chronic TBI as revealed by high resolution tensor magnetic resonance imaging [42]. Patients with a history of brain injury exhibit ventral thalamic atrophy, which correlates with impaired executive function, attention, and memory and learning deficits post-TBI [42]. Interestingly, cerebral blood flow to the thalamus is significantly reduced even by mild TBI and coincides with impairments in speech, learning and memory [43]. Taken together, impaired cognitive functions mediated by cortex, hippocampus, and thalamus may manifest in chronic TBI.

In the present study, cell proliferation was significantly affected by the cascade of events in chronic TBI, but while neuronal differentiation showed a trend of similar reduction, it did not reach statistical significance compared to sham control. These discrepant results may be due to the timing of histological analyses (acute vs. chronic), varying models of TBI (mild vs. moderate) and different animal species (mice vs. rat) [31], [32], [33], [37]. Despite the variability in experimental procedures and animal subjects, these studies, including ours, identify the susceptibility of newly formed cells within neurogenic niches, implicating the pivotal role of endogenous cells likely involved in the host reparative mechanism against TBI [37], [38].

Similar co-morbidity critical factors in the clinic, such as patient age, injury severity, and past medical history, influence the outcomes of TBI; however, neuroinflammation as a key cell death exacerbating factor appears consistent following brain injury [6], [8], [9], [10], [11], [16], [44], [45]. Upregulation of neuroinflammation in the present study was depicted by exacerbation of activated microglia cells in gray matter structures such as cortex, striatum, thalamus, and white matter including corpus callosum, fornix and cerebral peduncles. In the clinic, long-term microglia activation was visualized in vivo within the thalamus, putamen, occipital cortices, and white matter areas as the internal capsule, in patients exhibiting severe impairments in cognitive function up at least 11 months after moderate to severe TBI [44]. Consequently, microglial cells pose as a candidate target for abrogating cell death in an effort to develop novel anti-inflammation-based therapeutic modalities in TBI [4], [6], [7], [8], [9], [10].

The present findings of white matter changes, in chronic TBI, agree with previous reports demonstrating the negative influences of activated microglial cells after TBI [46], [47], [48]. White matter axonal injury is a very common feature in clinical setting after TBI, which accounts for impairments of cognitive function, and may result in high mortality rate [46], [47], [48]. Axonal degeneration, typical in white matter injury, interrupts the action potential throughout the cortex [46], [47], [49], and combined with overt activation of microglia cells in cortical and subcortical areas, may lead to impaired cell survival and cell proliferation in both immediate and remote areas of the impacted brain region [8], [9], [23], [50], [51]. In the present in vivo study, there were 70% and 34% decrements in cell proliferation in the SVZ and SGZ, respectively, in comparison to sham control. Our study suggests that cell proliferation is altered due to chronic neuroinflammation, but additional studies elucidating this cell death mechanism and its direct influence on impeding endogenous proliferation would be necessary to establish this point. Notwithstanding, these results advance the potential benefits of anti-inflammatory therapies during the chronic stage of TBI.

Taken together, these results indicate that while TBI is generally considered an acute injury, a chronic secondary cell death perturbation (i.e., neuroinflammation) and a diminished endogenous repair mechanism (i.e., cell proliferation) accompany the disease pathology over long-term. The recognition of long-term pathological disturbances associated with chronic inflammation and neuropsychological diseases suggests a vigilant follow-up monitoring of TBI patients in order to better manage the disease progression. A multi-pronged treatment targeting inflammatory and cell proliferative pathways may abrogate these chronic TBI pathological effects.

## Supporting Information

Figure S1 **Upregulation of MHCll+ activated microglia cells in gray matter in chronic TBI.** Results indicate that there is a clear exacerbation of activated microglia cells in ipsilateral side of subcortical gray matter regions in chronic TBI relative to contralateral side and sham control. After 8 weeks from initial TBI injury, asterisks denote significant upregulation on the volume of MHC II expressing cells in cortex, striatum, and thalamus. While contralateral side present an estimated volume of activated microglia cells similar to sham control animals. Photomicrographs correspond to representative gray matter in coronal sections stained with OX6 (MHC ll) in sham control and TBI. Arrows denote activated microglia cells. ***Upregulation of MHCll+ activated microglia cells in white matter in chronic TBI***. Results indicate that there is an upregulation of activated microglia cells after 8 weeks post TBI in proximal white matter areas. There is an upregulation of MHCll+ cells in the ipsilateral and contralateral side of corpus callosum relative to sham control. In contrast, upregulation of MHCll+ activated microglia cells in the cerebral peduncle and fornix is only present in the ipsilateral side as compared with the contralateral and sham control. There were no significant differences between contralateral side and sham control animals. Photomicrographs correspond to representative coronal sections stained with OX6 in sham control and TBI. Arrows denote activated microglia cells. Chronic TBI greatly upregulates the neuroinflammation in the thalamus expressing the highest upregulation of MHCll+ activated microglia cells, despite its distal subcortical location. Strong expression of MHCll+ activated microglia cells is also detected in the corpus callosum and striatum.
(TIF)

Figure S2 **Hippocampal CA3 cell loss and downregulation of cell proliferation.** H&E staining revealed a significant cell loss in the hippocampal CA3 region after chronic TBI (A). Ki67, (cell proliferation marker) revealed a significant chronic TBI-related decrease in the SVZ of cell proliferation only in the ipsilateral side relative to contralateral side and sham control animals. Contralateral measurements revealed that cell proliferation also decrease, but it does not show significant differences when compared with sham control animals. Also, Ki67 revealed a significant decrease in cell proliferation in the SGZ of the hippocampus in the ipsilateral side in compared to both contralateral side and sham control. Representative coronal sections stained with H&E in sham control and TBI are shown. Arrows denote neuronal cell loss in hippocampal CA3 area. In addition, representative images of SVZ and SGZ areas stained with Ki67 in sham control and TBI are shown. Arrows denote proliferating cells in SVZ and SGZ.
(TIF)

Figure S3 **Neuronal differentiation is not affected by chronic TBI.** DCX staining, neuronal differentiation marker revealed that there is not significant impairment in neuronal differentiation in either SVZ of the lateral ventricle, or the SGZ of the hippocampus relative to contralateral side and sham control animals. Representative coronal sections stained with DCX in sham control and TBI are shown. Arrows denote DCX positive cells in SVZ and in the SGZ.
(TIF)

Table S1 **MHC Class ll Cortex.**
(DOCX)

Table S2 **MHC Class ll Striatum.**
(DOCX)

Table S3 **MHC Class ll Thalamus.**
(DOCX)

Table S4 **MHC Class ll Corpus Callosum.**
(DOCX)

Table S5 **MHC Class ll Cerebral Peduncle.**
(DOCX)

Table S6 **MHC Class ll Fornix.**
(DOCX)

Table S7 **CA3 Neuronal Cell Loss.**
(DOCX)

Table S8 **Cell Proliferation SVZ.**
(DOCX)

Table S9 **Cell Proliferation SGZ.**
(DOCX)
