# Correction: Long-Term Upregulation of Inflammation and Suppression of Cell Proliferation in the Brain of Adult Rats Exposed to Traumatic Brain Injury Using the Controlled Cortical Impact Model

**DOI:** 10.1371/annotation/a04a7468-d105-42f3-ba47-263ea2864681

**Published:** 2013-08-09

**Authors:** Sandra A. Acosta, Naoki Tajiri, Kazutaka Shinozuka, Hiroto Ishikawa, Bethany Grimmig, David Diamond, Paul R. Sanberg, Paula C. Bickford, Yuji Kaneko, Cesar V. Borlongan

Author David Diamond should read David M. Diamond. This author is incorrectly associated with affiliation 3 only. His correct affiliations are 2 and 3:

2. James A. Haley Veterans Affairs Hospital, Tampa, Florida, United States of America

3. Department of Psychology, University of South Florida, Tampa, Florida, United States of America

There is a statement missing from the Competing Interests. The following statement should be considered part of this information:

The opinions expressed in this publication are those of the authors and not of the Department of Veterans Affairs or the US government. 

