# Supplementary figures and images for: Long-Term Upregulation of Inflammation and Suppression of Cell Proliferation in the Brain of Adult Rats Exposed to Traumatic Brain Injury Using the Controlled Cortical Impact Model
Source: PLoS One. 2013 Jan 3;8(1):e53376. doi: 10.1371/journal.pone.0053376 (PMC3536766; doi:10.1371/journal.pone.0053376)

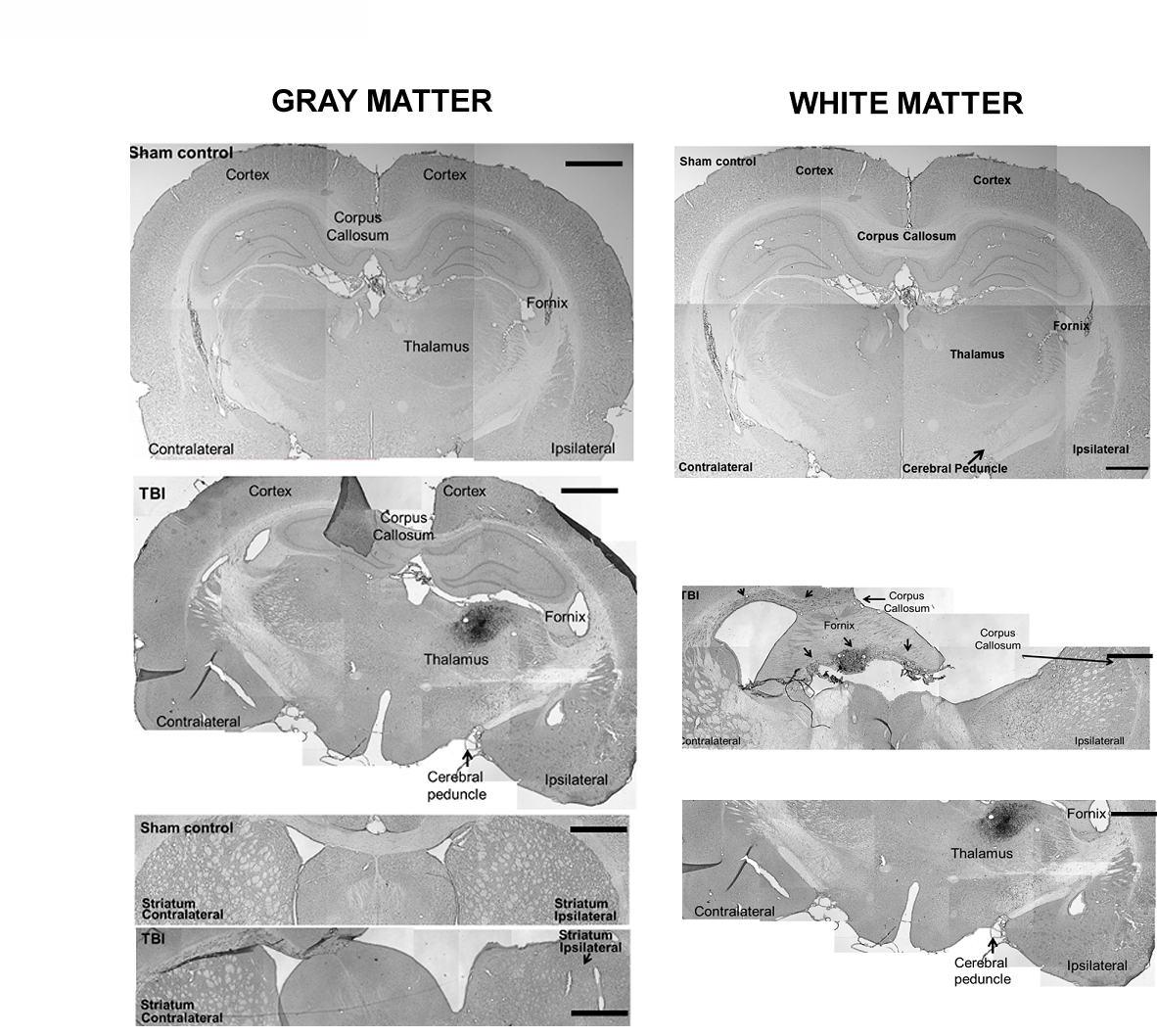

Supplement: Figure S1 — Upregulation of MHCll+ activated microglia cells in gray matter in chronic TBI. Results indicate that there is a clear exacerbation of activated microglia cells in ipsilateral side of subcortical gray matter regions in chronic TBI relative to contralateral side and sham control. After 8 weeks from initial TBI injury, asterisks denote significant upregulation on the volume of MHC II expressing cells in cortex, striatum, and thalamus. While contralateral side present an estimated volume of activated microglia cells similar to sham control animals. Photomicrographs correspond to representative gray matter in coronal sections stained with OX6 (MHC ll) in sham control and TBI. Arrows denote activated microglia cells. Upregulation of MHCll+ activated microglia cells in white matter in chronic TBI. Results indicate that there is an upregulation of activated microglia cells after 8 weeks post TBI in proximal white matter areas. There is an upregulation of MHCll+ cells in the ipsilateral and contralateral side of corpus callosum relative to sham control. In contrast, upregulation of MHCll+ activated microglia cells in the cerebral peduncle and fornix is only present in the ipsilateral side as compared with the contralateral and sham control. There were no significant differences between contralateral side and sham control animals. Photomicrographs correspond to representative coronal sections stained with OX6 in sham control and TBI. Arrows denote activated microglia cells. Chronic TBI greatly upregulates the neuroinflammation in the thalamus expressing the highest upregulation of MHCll+ activated microglia cells, despite its distal subcortical location. Strong expression of MHCll+ activated microglia cells is also detected in the corpus callosum and striatum. (TIF) [file pone.0053376.s001.tif]

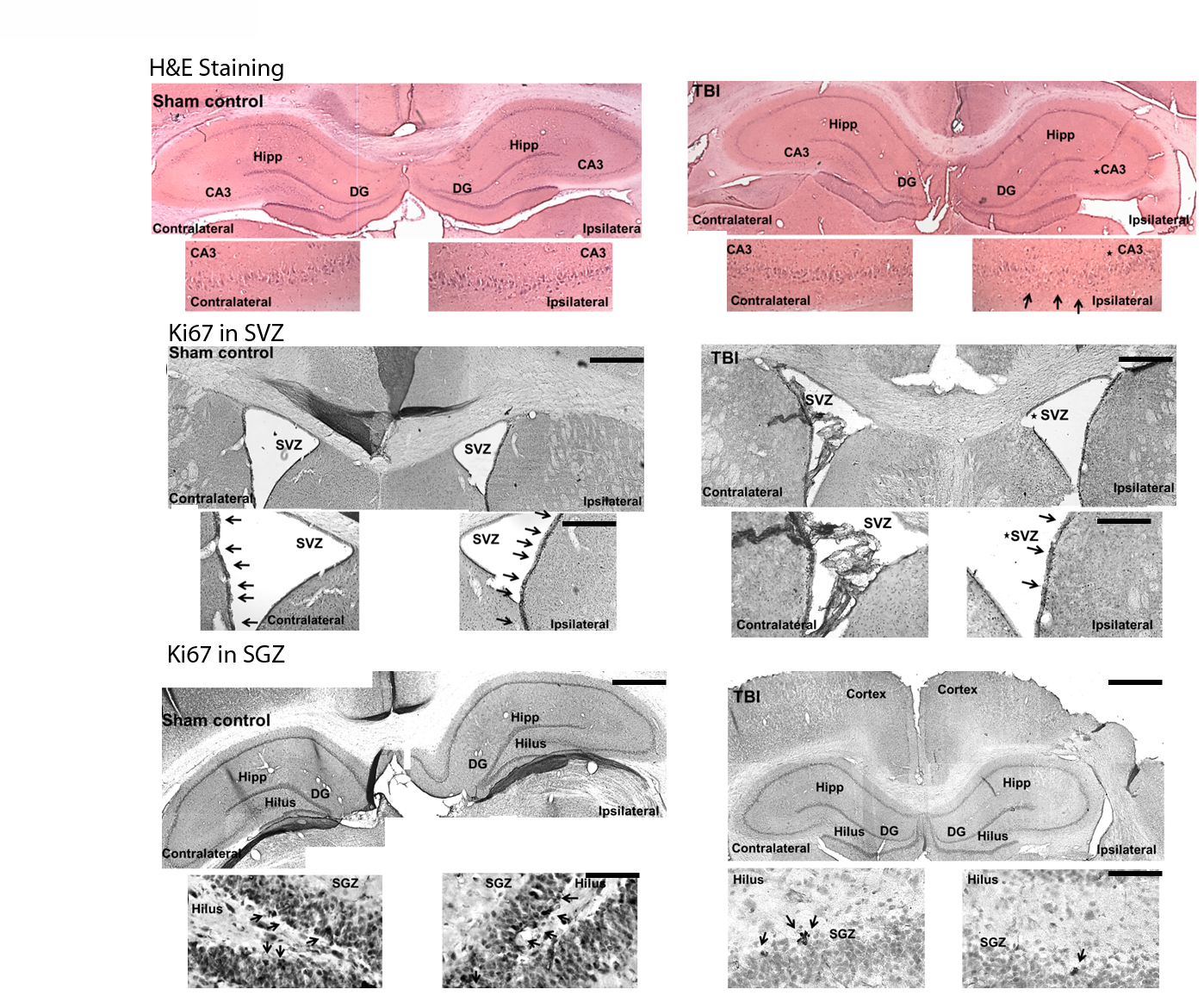

Supplement: Figure S2 — Hippocampal CA3 cell loss and downregulation of cell proliferation. H&E staining revealed a significant cell loss in the hippocampal CA3 region after chronic TBI (A). Ki67, (cell proliferation marker) revealed a significant chronic TBI-related decrease in the SVZ of cell proliferation only in the ipsilateral side relative to contralateral side and sham control animals. Contralateral measurements revealed that cell proliferation also decrease, but it does not show significant differences when compared with sham control animals. Also, Ki67 revealed a significant decrease in cell proliferation in the SGZ of the hippocampus in the ipsilateral side in compared to both contralateral side and sham control. Representative coronal sections stained with H&E in sham control and TBI are shown. Arrows denote neuronal cell loss in hippocampal CA3 area. In addition, representative images of SVZ and SGZ areas stained with Ki67 in sham control and TBI are shown. Arrows denote proliferating cells in SVZ and SGZ. (TIF) [file pone.0053376.s002.tif]

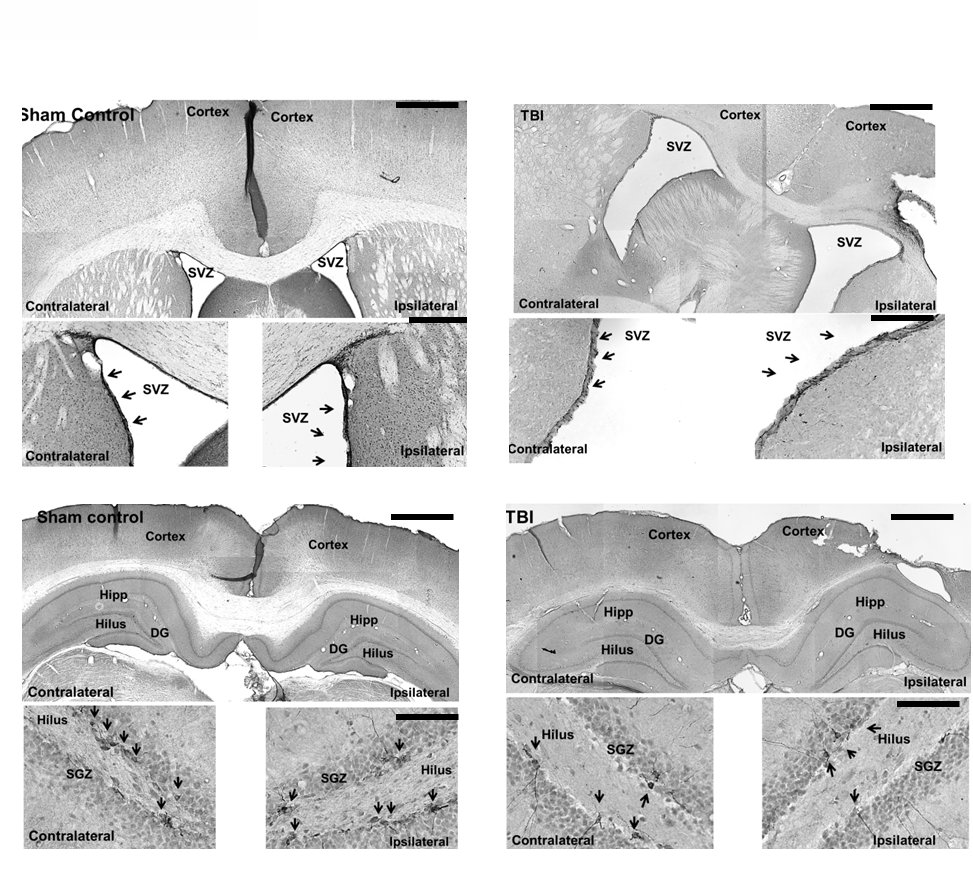

Supplement: Figure S3 — Neuronal differentiation is not affected by chronic TBI. DCX staining, neuronal differentiation marker revealed that there is not significant impairment in neuronal differentiation in either SVZ of the lateral ventricle, or the SGZ of the hippocampus relative to contralateral side and sham control animals. Representative coronal sections stained with DCX in sham control and TBI are shown. Arrows denote DCX positive cells in SVZ and in the SGZ. (TIF) [file pone.0053376.s003.tif]
