# Supplementary material for: Long-Term Upregulation of Inflammation and Suppression of Cell Proliferation in the Brain of Adult Rats Exposed to Traumatic Brain Injury Using the Controlled Cortical Impact Model
Source: PLoS One. 2013 Jan 3;8(1):e53376. doi: 10.1371/journal.pone.0053376 (PMC3536766; doi:10.1371/journal.pone.0053376)
Supplement: Table S1 — MHC Class ll Cortex. (DOCX) [file pone.0053376.s004.docx]

**Table S1: MHC Class ll Cortex**

| **Cortex (MHC II)** | **Sham Ipsi** | **Sham Contra** | **TBI ipsi** | **TBI contra** |
| --- | --- | --- | --- | --- |
| **Sham Ipsi** | **X** | **Ns p>0.05** | *****p<0.0001** | **Ns p>0.05** |
| **Sham Contra** | **Ns p>0.05** | **X** | *****p<0.0001** | **Ns p>0.05** |
| **TBI ipsi** | *****p<0.0001** | *****p<0.0001** | **X** | *****p<0.0001** |
| **TBI contra** | **Ns p>0.05** | **Ns p>0.05** | *****p<0.0001** | **X** |

Ipsi= Ipsilateral, Contra= contralateral, ns= not significant
