# Supplementary material for: Long-Term Upregulation of Inflammation and Suppression of Cell Proliferation in the Brain of Adult Rats Exposed to Traumatic Brain Injury Using the Controlled Cortical Impact Model
Source: PLoS One. 2013 Jan 3;8(1):e53376. doi: 10.1371/journal.pone.0053376 (PMC3536766; doi:10.1371/journal.pone.0053376)
Supplement: Table S3 — MHC Class ll Thalamus. (DOCX) [file pone.0053376.s006.docx]

**Table S3: MHC Class ll Thalamus**

| **Thalamus (MHC II)** | **Sham Ipsi** | **Sham Contra** | **TBI ipsi** | **TBI contra** |
| --- | --- | --- | --- | --- |
| **Sham Ipsi** | **X** | **ns** | ****p<0.01** | **ns** |
| **Sham Contra** | **ns** | **X** | ****p<0.01** | **ns** |
| **TBI ipsi** | ****p<0.01** | ****p<0.01** | **X** | ****p<0.01** |
| **TBI contra** | **ns** | **ns** | ****p<0.01** | **X** |

Ipsi= Ipsilateral, Contra= contralateral, ns= not significant
