# Supplementary material for: Long-Term Upregulation of Inflammation and Suppression of Cell Proliferation in the Brain of Adult Rats Exposed to Traumatic Brain Injury Using the Controlled Cortical Impact Model
Source: PLoS One. 2013 Jan 3;8(1):e53376. doi: 10.1371/journal.pone.0053376 (PMC3536766; doi:10.1371/journal.pone.0053376)
Supplement: Table S4 — MHC Class ll Corpus Callosum. (DOCX) [file pone.0053376.s007.docx]

**Table S4: MHC Class ll Corpus Callosum**

| **Corpus Callosum (MHC II)** | **Sham Ipsi** | **Sham Contra** | **TBI ipsi** | **TBI contra** |
| --- | --- | --- | --- | --- |
| **Sham Ipsi** | **X** | **ns** | ***p<0.05** | ***P<0.05** |
| **Sham Contra** | **ns** | **X** | ***p<0.05** | ***p<0.05** |
| **TBI ipsi** | ***p<0.05** | ***p<0.05** | **X** | **ns** |
| **TBI contra** | ***P<0.05** | ***p<0.05** | **ns** | **X** |

Ipsi= Ipsilateral, Contra= contralateral, ns= not significant
